# Illusion of competence: vision–language models provide confident but inaccurate explanations in cytological diagnostics

**DOI:** 10.1038/s41598-026-60372-6

**Published:** 2026-07-03

**Authors:** Ivan Kukuljan, Muhammed Furkan Dasdelen, Julia Schäfer, Michele Buck, Katharina S. Götze, Carsten Marr

**Affiliations:** 1 Computational Health Center & Helmholtz AI, Helmholtz Munich, Neuherberg, Germany; 2 School of Medicine and Health, Technical University of Munich, Munich, Germany; 3 Department of Medicine III, Hematology/Oncology, Technical University of Munich School of Medicine and Health, Munich, Germany; 4 Department of Medicine III, LMU Medizin, LMU Munich, Munich, Germany; 5 Department of Physics, Ludwig-Maximilians-Universität München, Munich, Germany; 6 German Cancer Consortium (DKTK), partner site Munich, Germany; 7 Munich Center for Machine Learning (MCML), Munich, Germany; 8 Bavarian Center for Cancer Research (BZKF), Munich, Germany

**Keywords:** Cancer, Computational biology and bioinformatics, Mathematics and computing, Medical research, Oncology

## Abstract

Large vision-language models (LVLMs) have shown impressive image-understanding capabilities across domains. However, their suitability for cytomorphological diagnostics remains unclear. Here, we systematically evaluated four state-of-the-art generalist LVLMs, GPT-4o, Gemini-2.0, Llama-3.2, and DeepSeek-VL2, and three biomedical LVLMs, LLaVA-Med, CONCH, and BiomedCLIP, across key cytomorphology benchmarks, including peripheral blood cell classification, morphology assessment, bone marrow cell classification, and cervical smear malignancy detection. Performance was assessed under zero-shot, few-shot, and fine-tuned settings. In zero-shot and few-shot evaluations, LVLMs performed poorly, often approaching random performance. In peripheral blood cell classification, GPT-4o achieved a zero-shot F1 score of only 0.22 ± 0.02 and a few-shot F1 score of 0.36 ± 0.03. Even after fine-tuning, GPT-4o was outperformed by a lightweight, dedicated hematology model. Beyond classification accuracy, we assessed interpretability and trustworthiness. Although LVLMs generated textual justifications, these often reflected textbook knowledge rather than the actual morphological features present in the cell images. Expert evaluation showed that 30% of explanations for misclassified cells were rated as poor or misleading. While LVLMs could segment cellular structures such as nuclei and granules, they failed to reliably identify the image regions relevant to their classification decisions. Our findings underscore three major limitations of current LVLMs in cytomorphology: (1) low diagnostic accuracy, (2) poor generalizability across domains, and (3) unreliable explainability. These results suggest that LVLMs require substantial improvement before they can be used for cell-type classification and morphology characterization in diagnostic settings. Purpose-built models remain the more effective and trustworthy choice.

**Supplementary Information:**

The online version contains supplementary material available at 10.1038/s41598-026-60372-6.

## Introduction

Cytology is the diagnostic evaluation of individual cells obtained from body fluids, while cytomorphology refers to the visual interpretation of cellular morphology for clinical decision-making. It is a cornerstone of hematology and gynecology, enabling cost-effective and minimally invasive diagnosis across diseases such as blood cancers, cervical cancer, and other neoplastic or inflammatory conditions^1,2^. In cytomorphological assessment, experts evaluate features such as a cell’s nuclear size and shape, chromatin pattern, nucleoli, cytoplasmic appearance, granularity, maturation stage, and the presence of atypical or malignant cells^3,4^. These observations are integrated to classify cell types, quantify abnormal populations, and support diagnosis. However, cytomorphology is challenging: diagnostically relevant differences can be subtle, abnormal cells may be rare, staining and preparation artifacts may alter appearance, and some cell types show overlapping morphology. As a result, interpretation requires substantial expertise, can be time-consuming, and is subject to interobserver variability. These challenges are further amplified by the global shortage of trained cytologists, even in high-income countries^5^.

Recent advances in AI have shown strong potential for cytomorphology-specific applications. Specialized models have achieved high performance in cervical smear screening^6–9^, blood cell classification^10–13^, and leukemia diagnosis^14–19^, with more recent work also developing foundation models for cytomorphology^20^. In parallel, large vision-language models (LVLMs) have emerged as general-purpose tools capable of processing multimodal data and have shown promise in medical image analysis, disease classification, visual question answering, and report generation^21–25^. Several studies have evaluated LVLMs in medical imaging, including endoscopy, chest X-ray, skin lesion analysis, ultrasound, mammography, radiology, dermatology, microscopy, pathology, and broader medical question-answering tasks^26–31^. However, despite this growing interest, the performance of LVLMs in cytomorphology remains largely unexplored. In particular, it is unclear whether generalist or medical-specific LVLMs can recognize subtle cellular features, distinguish morphologically similar cell types, and provide reliable explanations in this highly specialized diagnostic domain.

In this study, we systematically evaluated LVLMs on diverse cytomorphology tasks to answer three main questions: How well do generalist and medical-specific LVLMs perform on cytomorphology tasks? Is it more efficient to fine-tune LVLMs for cytomorphology-specific tasks, or is it better to develop dedicated AI models? Can we trust textual explanations these models provide? To answer these questions, we benchmarked generalist LVLMs, including GPT-4o, Gemini-2.0 Flash, Llama-3.2, and DeepSeek-VL2, as well as medical-specific models, including LLaVA-Med, CONCH, and BiomedCLIP. We assessed their zero-shot and few-shot performance across multiple cytomorphology datasets. Additionally, we fine-tuned GPT-4o for peripheral blood cell classification and compared its performance with a hematology foundation model.

## Methods

### Datasets & tasks

**Fig. 1:**
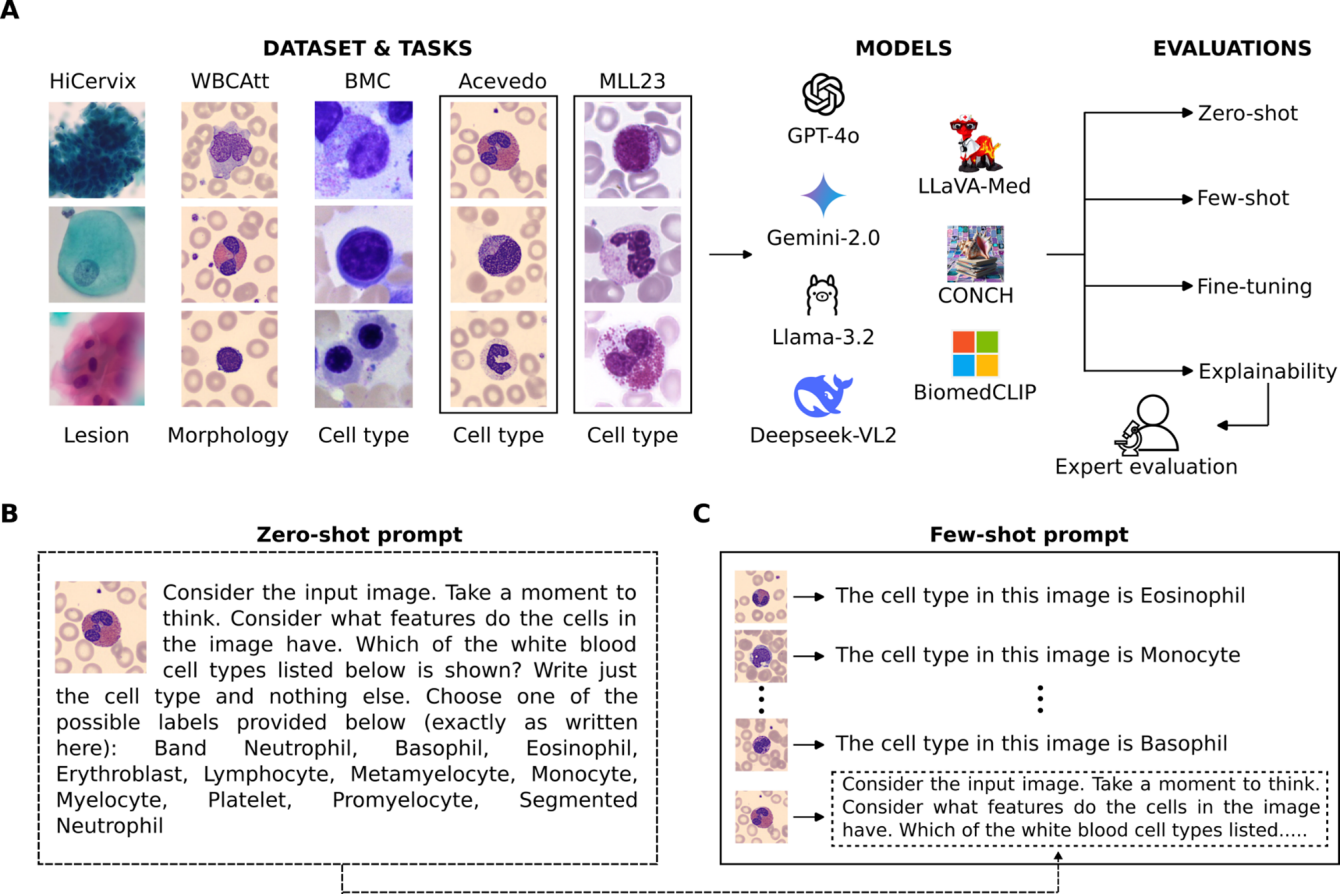
Systematic evaluation of Large Vision Language Models (LVLMs) in cytomorphology. (**A)** We used five cytomorphology and hematology datasets with respective tasks (HiCervix: cervical smear lesion classification; WBCAtt: white blood cell morphology classification; BMC: bone marrow cell type classification; Acevedo: peripheral blood cell type classification, MLL23: out-of-domain peripheral blood cell type classification) to assess generalist LVLMs (GPT-4o, Gemini-2.0, Deepseek-VL2, Llama-3.2) alongside medical vision-language models (LLaVA-Med, CONCH, BiomedCLIP) under zero-shot, few-shot, and fine-tuned conditions. (**B)** In zero-shot evaluation, the prompt consisted of a single image and a corresponding text query. (**C)** In few-shot evaluation, the prompt included an example image from each class with the corresponding label, followed by the input image and the text query.

To evaluate the performance of the most important LVLMs on cytomorphology tasks, we selected four data sets for classifying cell types or lesion types (peripheral blood cells, bone marrow cells and cervical cells) and one dataset quantifying peripheral blood cell morphologies (Fig. 1A):

- **HiCervix**^32^—The hierarchical dataset for cervical cytology classification comprises 40,229 cervical cells from 4496 whole slide images, categorized into 29 classes. HiCervix includes normal epithelial cells, infectious agents, and malignant cells.
- **Acevedo** et al.^33^ provide 17,092 white blood cell images from peripheral blood smears, labeled with 11 different cell type annotations.
- **BMC**^10^—The Bone Marrow Cytomorphology dataset is a collection of 171,373 white blood cell images from bone marrow smears collected from 945 patients. The cells were expert-labeled into 21 different cell types.
- **WBCAtt**^34^—The White Blood Cell dataset annotated with detailed morphological Attributes contains morphology annotations for 10,300 images from the Acevedo data set. Labels are provided for 11 fine-grained morphological attributes like nucleus shape, chromatin density, granularity, or cytoplasm texture.
- **MLL23**^35^**— The **Munich Leukemia Laboratory 2023 dataset was used only as an external test set for fine-tuned models. It includes over 40,000 expert annotated peripheral blood single cell images categorized into 18 classes.

For each dataset, we randomly sampled 50 images per class (or the maximum available samples if fewer than 50 images were present) as a test set across zero-shot, few-shot, and fine-tuning experiments. For few-shot learning, we selected an independent training set containing one image per class (Fig. 1C). For fine-tuning, only the Acevedo data set was used. We selected an independent subset of 200 images per class for training, and a separate validation set of 50 images per class. MLL23 was used exclusively for out-of-domain evaluations to assess the generalizability of fine-tuned models. The MLL23 test set contained only the classes present in Acevedo to ensure a fair comparison.

### Models

We evaluated four leading large vision-language models:

- **GPT-4o**^36^ is the Flagship model by OpenAI that can reason across audio, vision, and text in real time. The authors do not disclose the model’s architecture nor size.
- **Gemini-2.0-flash-exp**^37^ is Google Deepmind’s flagship vision language model. The authors do not disclose the model’s architecture nor size.
- **Llama-3.2-multimodal-11B**^38^ is the smaller of Meta AI’s vision language models with 11 billion weights, runnable on a single NVIDIA A100 GPU.
- **DeepSeek-VL2-small**^39^ belongs to the 2nd generation of DeepSeek vision language models. We evaluated the small versions with 2.8 billion weights.

We also evaluated three most prominent models specifically designed for the biomedical domain:

- **LLaVA-Med**^40^ is Microsoft’s vision language model for biomedical images. It has been trained on 15 million biomedical image-text pairs.
- **CONCH**^41^ is a state-of-the-art vision language foundation model for computational pathology, trained on over 1.17 million image-caption pairs.
- **BiomedCLIP**^42^ is a biomedical vision-language foundation model pretrained on the 15 million image-text pairs.
  Additionally, we included a hematology-specific model in our fine-tuning experiments for comparison with GPT-4o:
- **DinoBloom**^20^ is the state-of-the-art hematology foundation model. It is based on DINOv2 and trained on over 380,000 white blood cell images. We use the DinoBloom-S version with 22 million weights.

GPT-4o and Gemini-2.0 models are only available commercially through API calls, while the other models can be downloaded and run locally.

### Evaluations

**Zero-shot:** The model was presented with an image and asked to classify the cell it contained (Fig. 1B). Its prediction was then compared to the ground truth label from the dataset. The model was provided with a predefined list of labels from which it could choose. To increase the performance of the model, we instructed the model to take a moment to analyze the cell’s features before making a decision. To ensure a definitive classification, we removed ambiguous categories such as “not clear” or “not identifiable” from the list of possible answers. We evaluated zero-shot performance across all datasets. The prompt for the Acevedo dataset is shown in Fig. 1B. For the zero-shot CONCH and BiomedCLIP model evaluations, we compared embedding similarities between the image and the prompt text (see Supplementary Methods). The variance of the scores was computed by splitting the models’ answers into five non overlapping folds and computing the scores for each.

**Fig. 2:**
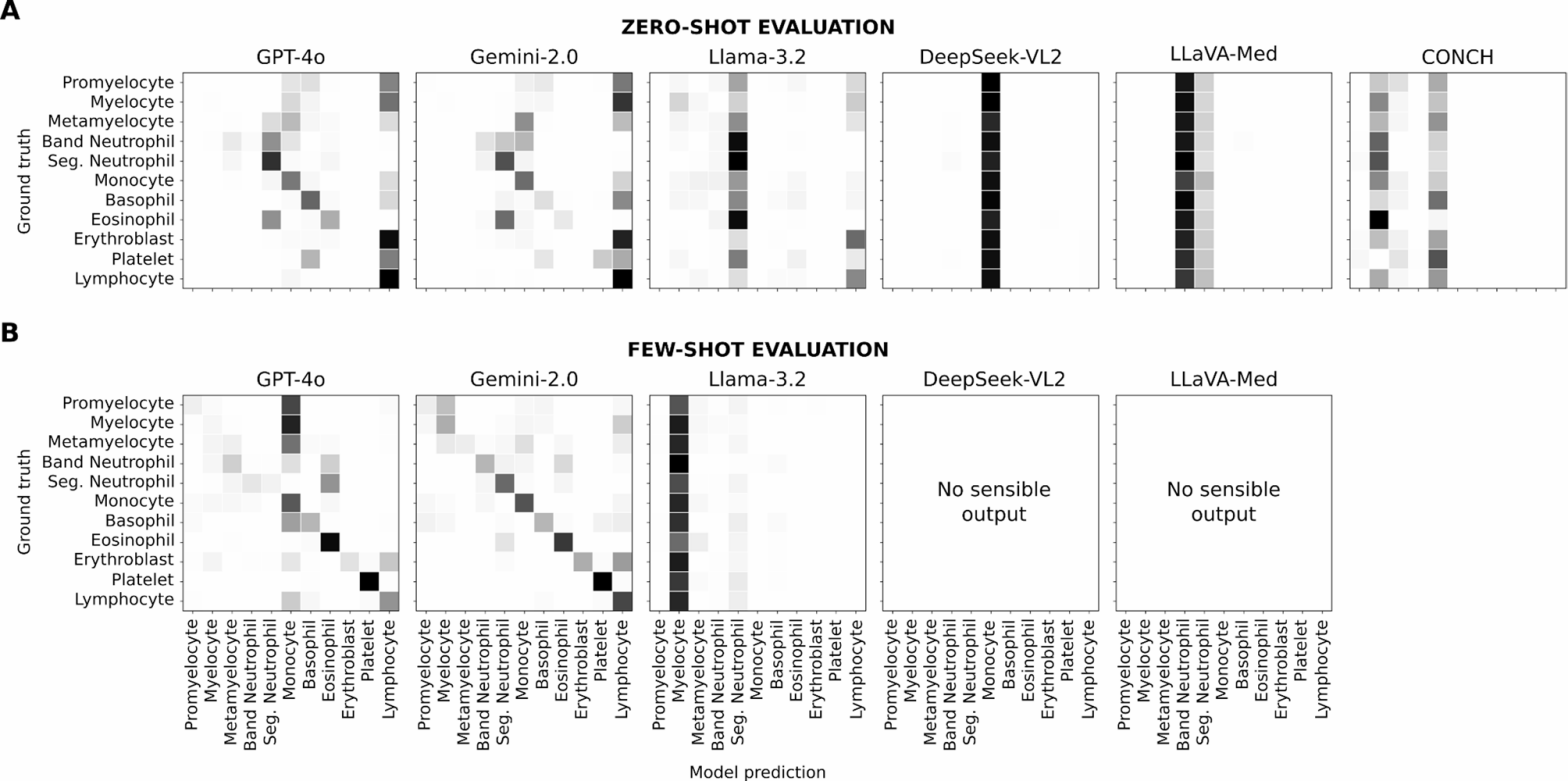
Large visual language models tend to classify cells into only a few cell types. Confusion matrices of models tested on the Acevedo test set with 50 images per cell type show high mis-classification in zero-shot evaluation (**A**) and moderate improvement for GPT-4o and Gemini-2.0 in few-shot evaluation (**B**). DeepSeek-VL2 and LLaVA-Med failed in few-shot evaluation: DeepSeek-VL2 generated erroneous text composed of random numbers and letters, while LLaVA-Med either gave no response or simply noted the presence of red blood cells and described their function.

**Few-shot:** The model was first shown one example image for each cell class in the dataset, along with a description of the corresponding class (Fig. 1C). It was then presented with an unknown image and asked to classify it. As in zero-shot evaluation, the model selected from a predefined list of possible answers. We evaluated few-shot performance across all datasets (Fig. 2).

**Fig. 3:**
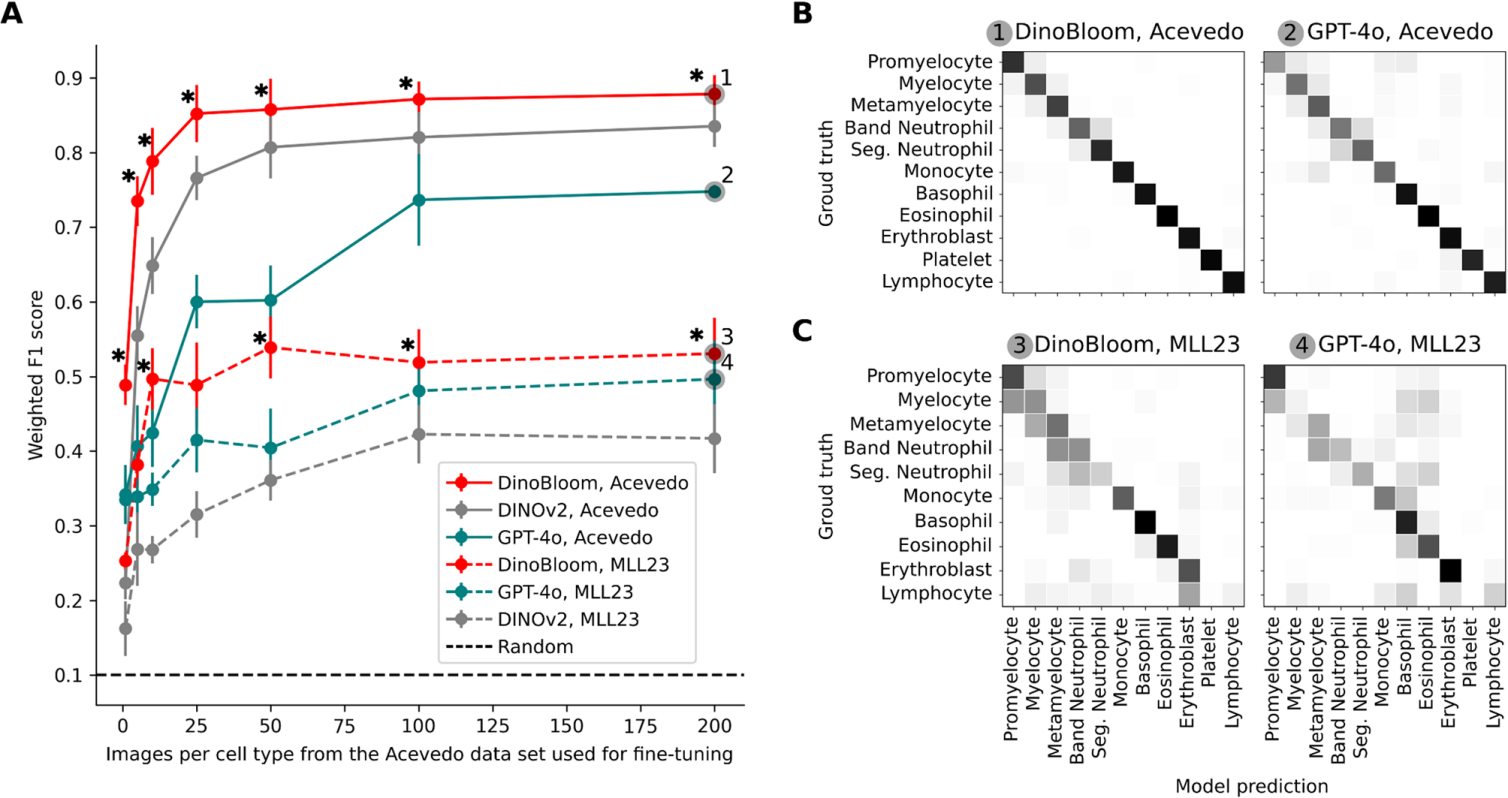
Cytology-specific and vision foundation models outperform large vision language models. **(A)** We fine-tuned GPT-4o, DINOv2 and DinoBloom models on a subset of the Acevedo dataset of different sizes (n = 1, 5, 10, 25, 50, 100, 200 images per cell type) and evaluated them on an independent Acevedo test set. DinoBloom and DINOv2 outperformed GPT-4o at any fine-tuning dataset size in terms of weighted F1 scores in-domain (Acevedo test set, solid lines) and out-of-domain (MLL23 test set, dashed lines). Confusion matrices of fine-tuned models evaluated on (**B**) Acevedo and (**C**) MLL23 proved superior performance of the cytology-specific DinoBloom model. * indicates statistical significance (p<0.05) between DinoBloom and GPT-4o performance.

**Fine-tuning:** To assess how effectively LVLMs could learn to interpret cytomorphology images, we fine-tuned GPT-4o via API access^43^ using the training subset of the Acevedo dataset (Fig. 3). The same test subset of Acevedo dataset was used as in the other evaluations (non overlapping with the train set). The number of fine-tuning samples per class was n = 1, 5, 10, 25, 50, 100, and 200. We compared the fine-tuned GPT-4o with the fine-tuned multilayer perceptron (MLP) on top of the DinoBloom model at each fine-tuning sample size. We also included MLP on top of the DINOv2—a non-medical-domain pretrained model—as a baseline. Separate models were trained for each dataset size. We specifically chose the Acevedo blood cell dataset for fine-tuning, as DinoBloom had not been trained on this dataset, ensuring a fair comparison between the two models. We also tested the fine-tuned models on the external test set, MLL23.

**Answer cleaning:** Although the models were instructed to provide a short answer containing the cell class only, they occasionally generated longer responses with explanations. To address this, we processed the answers through a chatbot once more, asking it to extract only the cell class from the chatbot’s answer. For consistency and reliability, GPT-4o was used for this, ensuring uniform conditions across all models.

### Explainability

We evaluated explainability of the four generalist models (GPT-4o, GPT-4o fine-tuned with 200 images per class, Gemini-2.0, and Llama-3.2) on the Acevedo data set.

**Fig. 4:**
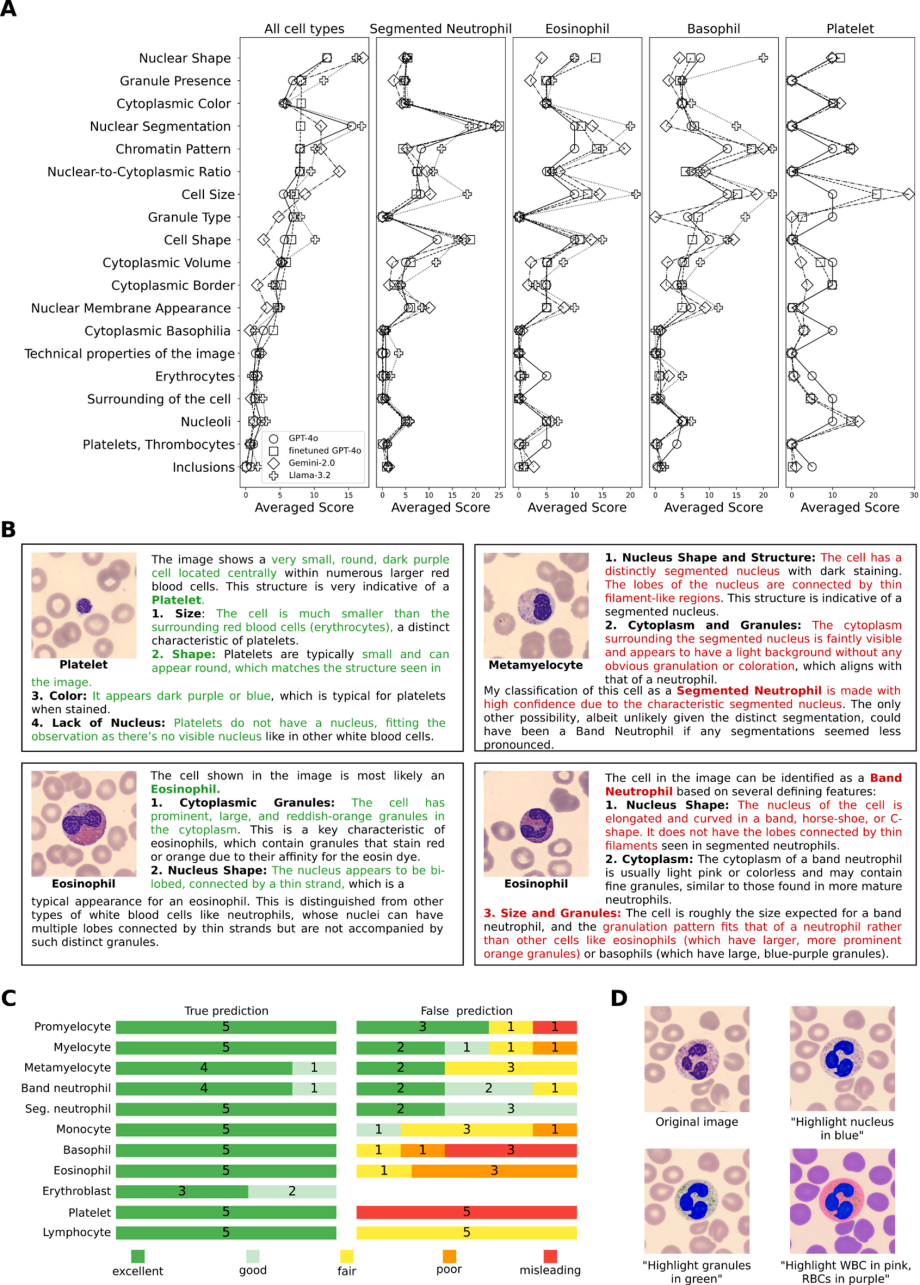
GPT-4o provides textbook explanations instead of truly interpreting cellular properties. **A.** Average self-reported feature-importance scores assigned by each model to 19 predefined morphological features. For each correctly classified image, the model was asked to distribute 100 points across the features according to their relevance for the classification decision. Scores were then averaged across correctly classified cells. **B.** Representative free-text explanations generated by fine-tuned GPT-4o. Examples include two correctly classified cells on the left and two incorrectly classified cells on the right. In incorrect predictions, the model often described features consistent with the predicted label rather than the actual morphology visible in the image. **C.** Expert cytomorphologist evaluation of fine-tuned GPT-4o explanations. Explanations for correctly classified cells were mostly rated as excellent, whereas 30% of explanations for misclassified cells were rated as poor or misleading. **D.** ChatGPT-4o segments cellular components successfully when clear nuclei and granules are present. Failure cases are shown in Supplementary Fig. 1.

**Quantitative feature importance:** We presented the model with all 549 cells in the Acevedo test set, one by one, and asked it to classify the cell. Then it was presented with a list of 19 morphological features (see Fig. 4A) and asked to assign them a score on how relevant they were for the classification with the following prompt:

"Consider the input image. […] Which of the white blood cell types listed below is shown? […] Now consider the cell features listed below. Think how much each of them contributed to your cell classification decision that you made above. Next to each feature, write an importance score how much the feature was important for your classification decision. The scores should be float numbers. All the scores together should sum to 100.

Cell Shape, Cell Size, Nuclear Shape, Nuclear Segmentation, Nuclear-to-Cytoplasmic Ratio, Nuclear Membrane Appearance, Nucleoli, Chromatin Pattern, Cytoplasmic Volume, Cytoplasmic Color, Cytoplasmic Border, Granule Presence, Granule Type, Inclusions (Presence of Auer rods, Döhle bodies, or other cytoplasmic inclusions), Cytoplasmic Basophilia, Erythrocytes, Platelets, Thrombocytes, Surrounding of the cell, Technical properties of the image (resolution, light, noise, etc.). Are there any other features that you consider important for the classification decision? If yes, write them below."

We averaged the scores for all the cells and for individual cell types (Fig. 4A) for those answers where the predicted label was true.

**Model explanation:** For the Acevedo test set, we asked the model to classify a cell and then provide a free-text explanation of the decision (Fig. 4B, C) using the following prompt:

“Consider the input image. […] Which of the white blood cell types listed below is shown? […] Explain in detail your decision and the reasoning that lead you to the decision. Which parts of the cells and features did you consider? How certain are you about your classification? Which other labels could be correct? Why did you choose this label in the end?”

**Expert evaluation:** We randomly selected 10 images per cell type (5 correctly and 5 incorrectly classified images), along with their corresponding explanations generated by the fine-tuned GPT-4o. All images were de-identified and presented to an expert cytomorphologist (M.B.) without indicating whether they were correctly or incorrectly classified. The expert was first asked to classify each image independently and then provided with the corresponding GPT-4o-generated explanation. The expert rated each explanation based on how well it aligned with the cell’s morphological characteristics, using a 5-point scale: 1 – excellent, 2 – good, 3 – fair, 4 – poor, 5 – misleading or completely incorrect.

**Computer vision:** To assess the model’s understanding of cellular components, we asked it to highlight the nucleus in blue, granules in green, the entire white blood cell in pink, and red blood cells in purple, one at a time. We also asked the model to highlight parts of the cell relevant for the cell classification. API access could not be utilized, as it currently lacks image generation capabilities. Gemini-2.0 failed to generate meaningful images. Therefore, we focused our evaluation on GPT-4o.

### Statistical analysis

In fine-tuning experiments, we performed a paired bootstrap analysis with 20,000 different random re-samples of the test set. We computed the differences in weighted F1 scores between fine-tuned DinoBloom and GPT-4o. p values lower than 0.05 were considered statistically significant.

## Results

We evaluated four generalist large vision-language models (LVLMs) and three medical hematology-specific LVLMs on four different data sets (Fig. 1) and quantified their performance using the weighted F1 score (Table 1). As a reference, we considered the performance of models reported in the original papers of the respective dataset. Given that the LVLMs mostly performed much worse than SOTA, we also compared them to random guessing models. Weighted F1 performance of a random model was calculated based on class distribution. LVLMs perform very poorly on zero-shot cell classification tasks, often yielding results similar to random performance and consistently far below the performance of models specifically designed for these tasks. For instance, the highest LVLM score for bone marrow cell classification (BMC) was 0.09 ± 0.02 (achieved by Gemini-2.0), compared to 0.05 for random guessing, and 0.75 for the model reported in the original study (Table 1). Similarly, for the cervical smear dataset, Gemini-2.0 achieved the best score of 0.06 ± 0.01, while the random model scores 0.04, and the reference model reached an average accuracy of 0.83. Notably, GPT-4o frequently stated that it did not know the answer to this task, likely due to the scarcity of publicly available cervix cytomorphology datasets.

**Table 1:** Large visual language models (LVLMs) remain substantially below published benchmarks, performing close to random models.

	HiCervix	Acevedo	BMC	WBCAtt
**Random**	0.04	0.09	0.05	0.49
Zero-shot performance (weighted F1)				
Gpt-4o	0.03 ± 0.02	0.22 ± 0.02	0.08 ± 0.02	0.35 ± 0.03
Gemini-2.0	**0.06 ± 0.01**	**0.24 ± 0.04**	**0.09 ± 0.02**	**0.49 ± 0.02**
Llama-3.2	0.02 ± 0.01	0.10 ± 0.03	0.04 ± 0.02	0.36 ± 0.02
Deepseek-VL2	0.01 ± 0.00	0.02 ± 0.00	0.01 ± 0.00	0.26 ± 0.01
LLaVA-Med	0.02 ± 0.01	0.02 ± 0.01	0.02 ± 0.01	0.38 ± 0.01
CONCH	0.01 ± 0.00	0.03 ± 0.01	0.03 ± 0.01	0.26 ± 0.02
BiomedCLIP	0.03 ± 0.01	0.08 ± 0.01	0.03 ± 0.01	0.38 ± 0.02
Few-shot performance (weighted F1)				
Gpt-4o	0.09 ± 0.01	0.36 ± 0.03	0.12 ± 0.02	0.64 ± 0.03
Gemini-2.0	0.15 ± 0.02	0.59 ± 0.09	0.19 ± 0.02	0.68 ± 0.01
Llama-3.2	0.01 ± 0.01	0.04 ± 0.01	0.02 ± 0.01	0.39 ± 0.02
Original publication	^**α**^**0.83**^32^	^**β**^**0.96**^13^	^**γ**^**0.75**^10^	^**δ**^**0.91**^34^

The weighted F1 scores achieved by the LVLMs are far from the state-of-the-art results from literature (last row) and can thus not be considered competitive. Interestingly, their performance is close to random guessing. Few-shot learning doubles the scores for Gemini-2.0 and GPT-4o in almost all tasks, however, they are still far below the state of the art. Mean and standard deviation were computed by splitting the test set into five balanced folds. We compare the following data sets: HiCervix^32^—Hierarchical Dataset Cervical Cytology Classification, Acevedo^33^—peripheral blood cells data set, BMC^10^—Bone Marrow Cytomorphology dataset, WBCAtt^34^—White Blood Cell Dataset Annotated with Detailed Morphological Attributes, Results from the original publication are α**:** averaged accuracy, β**:** accuracy, γ: weighted F1 score, and δ: averaged macro F1 score, as listed in the original publication.

The confusion matrices (Fig. 2A) for zero-shot blood cell classification on the Acevedo data set show that current LVLMs tend to misclassify cells into a few dominant classes: GPT-4o, Gemini-2.0, and Llama-3.2 predominantly assigned cells to lymphocytes or segmented neutrophils, while LLaVA-Med consistently classified cells as bands or segmented neutrophils. The computational pathology model CONCH mostly classified cells as segmented neutrophils or myelocytes.

Few-shot learning improved the performance, nearly doubling the scores for GPT-4o and Gemini-2.0 across most tasks (Table 1). For instance, Gemini-2.0 bone marrow cell classification score increased to 0.19 ± 0.02, while its cervical smear classification score improved to 0.15 ± 0.02. However, the scores remained closer to random model performance and still far from the state-of-the-art results. Figure 2B visualizes the improved confusion matrices for blood cell classification in the Acevedo data. Few-shot learning led to more diagonally aligned confusion matrices for GPT-4o and Gemini-2.0, indicating better classification. Llama-3.2 performed worse in few-shot learning, yielding even lower accuracy than in the zero-shot setting. DeepSeek-VL2 and LLaVA-Med did not produce sensible output in a few-shot setting. DeepSeek-VL2 generated erroneous text composed of random numbers and letters (for example “11–12-1–1-1–1-1–1-1–1-3–1-1–1-3–1-1…”), while LLaVA-Med either gave no response or simply noted the presence of red blood cells and described their function (for example “RBCs (red blood cells) are the most common cell type in the blood. They are responsible for transporting oxygen to the body’s tissues and organs.”).

We fine-tuned GPT-4o and DinoBloom on the Acevedo blood cell classification task (a data set which none of the models had been pretrained on), varying the number of training images per class, and evaluated performance on a left out test set (Fig. 3A). Model performance improved rapidly with increasing dataset size: With 10 images per class, GPT-4o’s weighted F1 score rose from 0.22 ± 0.02 (no fine-tuning) to 0.55 ± 0.06, further improving to 0.65 ± 0.05 with 50 images per class, to 0.74 ± 0.04 with 100 images per class, and plateauing from there on (Fig. 3A). Interestingly, a simple MLP trained on DINOv2 and DinoBloom models learns significantly faster and better than GPT-4o. In particular the hematology foundation model DinoBloom^20^ excels, achieving scores of 0.79, 0.86, and 0.87 for 10, 50, and 100 images per class, significantly outperforming GPT-4o at every training size with p < 10^–4^ (Fig. 3A, Supplementary Table 1; paired bootstrap test). The confusion matrices for fine-tuned GPT-4o and DinoBloom reveal that misclassifications primarily occur between morphologically similar cell types, such as myelocytes vs. metamyelocytes or segmented vs. band neutrophils—categories that are inherently difficult to distinguish (Fig. 3B).

To assess model generalizability, we evaluated the fine-tuned GPT-4o and DinoBloom on the out-of-distribution MLL23 test set (Fig. 3A, C). Notably, both models were fine-tuned for Acevedo cell classification. While GPT-4o was better at n = 1 and models had a similar performance at n = 5, 25, DinoBloom, with an MLP head, consistently and significantly outperformed GPT-4o at other values of n with p < 10^–4^ (Supplementary Table 2, paired bootstrap test). At n = 200, DinoBloom achieved a weighted F1 score of 0.54, compared to 0.50 for the fine-tuned GPT-4o. The zero-shot GPT-4o classification performance on MLL23 was low at 0.19, only slightly above the random baseline of 0.10. Confusion matrices reveal that misclassifications mostly occurred between cell types that are morphologically close to each other (Fig. 3C).

To assess the degree of explainability provided by LVLMs, we examined whether they could identify the morphological features underlying their blood cell classifications. For each cell in the test set, models were asked to first classify the image and then assign importance scores to 19 predefined morphological features, such as nuclear shape, chromatin pattern, cytoplasmic appearance, and granularity (see Methods for details and prompts). The models produced cell-type-specific feature-importance patterns, with moderate differences between models (Fig. 4A). For example, all models consistently assigned high importance to nuclear shape. Some of the highlighted features were biologically plausible and aligned with expert reasoning, such as nuclear segmentation for segmented neutrophils, cell size for platelets, and chromatin pattern and cell size for eosinophils. However, the models also missed important expert-defined features. For instance, granule presence and granule type, which are central for recognizing eosinophils, were not consistently identified as important. In addition, nucleoli were considered important for platelets, although platelets lack both nuclei and nucleoli. These findings suggest that LVLMs can sometimes report relevant morphological cues, but their explanations remain incomplete and do not fully capture expert cytomorphological reasoning.

We next asked an expert cytomorphologist to assess free text explanations for 10 images per cell type (5 correctly and 5 incorrectly classified by the best performing LVLM, the fine-tuned GPT-4o, see Fig. 4B for examples) on the following scale: 1—excellent, 2—good, 3—fair, 4—poor, 5—misleading. Explanations for correctly classified cell images received an average score of 1.1 ± 0.3 (mean ± s.d., n = 55), while wrongly classified images received a considerably lower score of 2.8 ± 1.4, with 30% of explanations being rated as poor or misleading. Explanations for incorrect classifications often missed or misinterpreted key white blood cell features (Fig. 4B). For example, the model described a kidney-shaped, unsegmented nucleus as segmented in a misclassified metamyelocyte, and incorrectly characterized the cytoplasmic granulation of a misclassified eosinophil as fine and non-eosinophilic. These examples suggest that the model often generated textbook-like descriptions of the predicted cell type rather than explanations grounded in the actual morphological features visible in the image. To evaluate GPT-4o’s vision capabilities, we asked the model to perform step-by-step segmentations (Fig. 4D). GPT-4o demonstrated a good understanding of cellular components by correctly identifying and segmenting relevant structures, but occasionally struggled with cell types such as platelets, basophils and erythroblasts (Supplementary Fig. 1).

In the free text answers, the models were also asked how certain they were about their classification decision. Their answers were evaluated using GPT-4o and sorted into the following confidence scores (conf): 1—very confident, 2—confident, 3—neutral, 4—non-confident, 5—very non-confident. We computed the correlation coefficient (corr) between the confidence scores and correctness of the predicted label. We also computed mean and standard deviation of the confidence scores separately for the answers with correctly predicted labels and those with wrong labels. The results for the top performing models are: GPT-4o: corr = 0.03, conf_correct_ = 2.05 ± 0.62, conf_wrong_ = 2.08 ± 0.62, fine-tuned GPT-4o (n = 200): corr = 0.20, conf_correct_ = 1.63 ± 0.54, conf_wrong_ = 1.87 ± 0.47, gemini-2.0: corr = 0.05, conf_correct_ = 1.82 ± 0.55, conf_wrong_ = 1.87 ± 0.44, Llama-3.2: corr = 0.002, conf_correct_ = 2.14 ± 0.79, conf_wrong_ = 2.14 ± 0.83. For the fine-tuned GPT-4o where we also had an expert evaluation of the answers available, we computed the correlation coefficient between the model’s confidence and the score assigned by the expert: corr = -0.01. We see that models were generally confident about their decisions even when the predicted classes were wrong. Llama-3.2 was less confident than the other models but independent whether the predicted label was correct or wrong. The only model that showed at least a tiny bit of correlation with the correctness of the predicted label and its confidence was fine-tuned GPT-4o, however 0.20 is still rather low. This came in combination with an even increased overall confidence of the model.

## Discussion

Our results demonstrate that current LVLMs underperform in fundamental tasks of cytomorphology such as cell classification. Across four clinically relevant cytomorphology tasks, generalist LVLMs often produced results close to random guessing, particularly in zero-shot settings. Models frequently defaulted to dominant classes such as segmented neutrophils and lymphocytes, which together represent over 75% of white blood cells in peripheral blood^44^ (Supplementary Table 3). This suggests reliance on prior probability rather than true morphological interpretation. Few-shot prompting improved performance, but it remained well below that of cytology-specific models reported in the literature. The observed limitations likely reflect a lack of cytomorphology-specific data in the training corpora of LVLMs. DeepSeek-VL2 and LLaVA-Med failed in the few-shot setting indicating that these smaller models are not tuned well to processing multiple image inputs, likely due to a limited context length.

Fine-tuning GPT-4o yielded notable gains, but performance remained consistently inferior to a simple multilayer perceptron (MLP) trained on top of a cytomorphology-specific foundation model. Importantly, the cytology-specific model not only achieved higher accuracy but also converged faster and required less computational effort. The model reached performance plateaus with as few as 100 images per class, aligning with prior reports^45^. Our findings are consistent with those of Jiang et al.^26^, who reported below-baseline performance of LVLMs on endoscopy images, chest X-rays, and skin lesions.

We also investigated explainability—a critical requirement for clinical deployment. Although LVLMs could generate plausible justifications, their explanations for misclassified cases revealed a significant limitation: they tended to provide generic, textbook-level descriptions rather than specific morphological reasoning grounded in the actual image features. This suggests weak integration between visual and language modalities, likely stemming from insufficient multimodal training on cytomorphology data. Future research should explore whether expanded training datasets or enhanced reasoning architectures can address these limitations and achieve the level of precise, image-specific explanations necessary for clinical practice.

In summary, current LVLMs remain unsuitable for fundamental tasks of cell type and morphology classification in cytomorphological diagnostics. Their limitations in performance, generalization, and interpretability significantly lag behind purpose-built, cytology-specific models. For clinical institutions and AI companies alike, investing in specialized models trained on carefully curated datasets might offer a more effective and cost-efficient path forward. Our findings underscore three critical requirements for clinical readiness: improved multimodal alignment to better integrate visual and textual understanding, inclusion of cytology-specific data during pretraining, and rigorous evaluation protocols that reflect real-world diagnostic challenges. Until these fundamental issues are addressed, LVLMs cannot be considered viable tools for clinical diagnostic use. However, in their current state, they may already be useful in educational settings or as language models alone.

## Supplementary Information

Below is the link to the electronic supplementary material.

Supplementary Material 1.

## Data Availability

The data used in this study is publicly available. HiCervix: https://zenodo.org/records/11081816 WBCAtt: https://github.com/apple2373/wbcatt/tree/main/submission Acevedo: https://data.mendeley.com/datasets/snkd93bnjr/1 BMC: https://www.cancerimagingarchive.net/collection/bone-marrow-cytomorphology_mll_helmholtz_fraunhofer/ MLL23: https://zenodo.org/records/14277609

## Code availability

The code used in this study is available at: https://github.com/marrlab/vlm_cytomorphology_eval
