# Supplementary material for: Illusion of competence: vision–language models provide confident but inaccurate explanations in cytological diagnostics
Source: Sci Rep. 2026 Jul 3;16:20526. doi: 10.1038/s41598-026-60372-6 (PMC13332019; doi:10.1038/s41598-026-60372-6)
Supplement: Supplementary file 1 — Supplementary Material 1. [file 41598_2026_60372_MOESM1_ESM.pdf]

Illusion of competence: Vision–language models provide confident but inaccurate explanations in cytological diagnostics

Ivan Kukuljan[1#], Muhammed Furkan Dasdelen[1#], Julia Schäfer[1,2], Michele Buck[3], Katharina S. Götze[3], Carsten Marr[1,4,5,6,7*]

[1]Institute of AI for Health, Computational Health Center, Helmholtz Munich, Ingolstädter Landstr. 1, 85764 Neuherberg, Germany
[2]Technical University Munich, School of Medicine and Health, Ismaninger Str. 22, 81675 München, Germany
[3]Klinikum rechts der Isar, III. Medizinische Klinik, Technische Universität München, Ismaninger Str. 22, 81675 München, Germany
[4]Department of Medicine III, Ludwig-Maximilian-University Hospital, Munich, Germany
[5]Department of Physics, University of Munich, Munich, Germany
[6]DKTK, German Cancer Consortium, Munich, Germany
[7]Munich Center for Machine Learning (MCML), Munich, Germany
# Shared first authorship, *Corresponding author: carsten.marr@helmholtz-munich.de

Supplementary Material

Prompts

Zero-shot and few-shot evaluation

Except for the CONCH and BiomedCLIP models, we used prompts to get answers from LVLMs. For each dataset, we used following question prompts in zero-shot and few-shots settings:

HiCervix:

```
"Consider the input image. Take a moment to think. Consider what
features do cells in the image have. Which of the  types listed below is
shown?
    Write just the cell type and nothing else. Choose one of the
possible labels provided below (exactly as written here):
    Normal cell
    Endocervical cell
    Repair cell
    Metaplasia cell
    Pseudokoilocytes by glycogen
    Atrophy
    Endometrial cell
    Hyperchromatic crowded groups
    Atypical squamous cells of undetermined significance
    Low-grade squamous intraepithelial lesion
    Atypical squamous cells, cannot exclude high-grade squamous
intraepithelial lesion
```

```
    High-grade squamous intraepithelial lesion
    Squamous cell carcinoma
    Atypical glandular cell
    Atypical glandular cell- not otherwise specified
    Atypical glandular cell- favor neoplastic
    Adenocarcinoma
    Atypical glandular cell (endocervical cell)- not otherwise specified
    Atypical glandular cell (endometrial cell)- not otherwise specified
    Adenocarcinoma of endocervical cell
    Adenocarcinoma of endometrial cell
    Fungal organisms morphologically consistent with Candida spp.
    Bacteria morphologically consistent with Actinomyces spp.
    Trichomonas vaginalis
    Cellular changes consistent with herpes simplex virus
    Coccobacilli/Shift in flora suggestive of bacterial vaginosis"
```

Acevedo:

```
"Consider the input image. Take a moment to think. Consider what
features do the cells in the image have. Which of the white blood cell
types listed below is shown?
    Write just the cell type and nothing else. Choose one of the
possible labels provided below (exactly as written here):
    Band Neutrophil
    Basophil
    Eosinophil
    Erythroblast
    Lymphocyte
    Metamyelocyte
    Monocyte
    Myelocyte
    Platelet
    Promyelocyte
    Segmented Neutrophil"
```

BMC:

```
"Consider the input image. Take a moment to think. Consider what
features do the cells in the image have. Which of the white blood cell
types listed below is shown?
    Write just the cell type and nothing else. Choose one of the
possible labels provided below (exactly as written here):
    Abnormal eosinophil
    Artefact
    Basophil
    Blast
    Erythroblast
```

```
    Eosinophil
    Faggott cell
    Hairy cell
    Smudge cell
    Immature lymphocyte
    Lymphocyte
    Metamyelocyte
    Monocyte
    Myelocyte
    Band neutrophil
    Segmented neutrophil
    Other cell
    Proerythroblast
    Plasma cell
    Promyelocyte"
```

WBCAtt:

```
label: "Consider the input image. Take a moment to think. Consider what
features do the cells in the image have. Which of the white blood cell
types listed below is shown? Write just the cell type and nothing else.
Choose one of the possible labels provided below (exactly as written
here):
    Neutrophil
    Eosinophil
    Basophil
    Lymphocyte
    Monocyte",
```

```
    cell_size: "Consider the white blood cell shown in the input image.
Take a moment to think. What is the size of the white blood cell? Choose
one of the possible labels provided below (exactly as written here):
    big
    small",
```

```
    cell_shape: "Consider the white blood cell shown in the input image.
Take a moment to think. What is the shape of the white blood cell?
Choose one of the possible labels provided below (exactly as written
here):
    round
    irregular",
```

```
nucleus_shape: "Consider the white blood cell shown in the input image.
Take a moment to think. What is the shape of the nucleus of the white
```

```
blood cell? Choose one of the possible labels provided below (exactly as
written here):
    unsegmented-band
    unsegmented-round
    segmented-multilobed
    segmented-bilobed
    irregular
    unsegmented-indented",
```

```
  nuclear_cytoplasmic_ratio: "Consider the white blood cell shown in the
input image. Take a moment to think. What is the ratio of the nucleus to
the cytoplasm of the white blood cell? Choose one of the possible labels
provided below (exactly as written here):
    low
    high",
```

```
 chromatin_density: "Consider the white blood cell shown in the input
image. Take a moment to think. What is the chromatin density of the
white blood cell? Choose one of the possible labels provided below
(exactly as written here):
    densely
    loosely",
```

```
 cytoplasm_vacuole: "Consider the white blood cell shown in the input
image. Take a moment to think. Does the white blood cell have a
cytoplasmic vacuole? Choose one of the possible labels provided below
(exactly as written here):
    no
    yes",
```

```
  cytoplasm_texture: "Consider the white blood cell shown in the input
image. Take a moment to think. What is the texture of the cytoplasm of
the white blood cell? Choose one of the possible labels provided below
(exactly as written here):
    clear
    frosted",
```

```
cytoplasm_colour: "Consider the white blood cell shown in the input
image. Take a moment to think. What is the colour of the cytoplasm of
the white blood cell? Choose one of the possible labels provided below
(exactly as written here):
    light blue
```

```
    blue
    purple blue",
```

```
granule_type: "Consider the white blood cell shown in the input image.
Take a moment to think. What is the type of the granules in the white
blood cell? Choose one of the possible labels provided below (exactly as
written here):
    small
    round
    coarse
    nil",
```

```
granule_colour: "Consider the white blood cell shown in the input image.
Take a moment to think. What is the colour of the granules in the white
blood cell? Choose one of the possible labels provided below (exactly as
written here):
    pink
    purple
    red
    nil",
```

```
granularity: "Consider the white blood cell shown in the input image.
Take a moment to think. Does the white blood cell have granules? Choose
one of the possible labels provided below (exactly as written here):
    yes
    no "
```

For few-shot experiments, we presented an example from each class before asking the same question as in the zero-shot prompt.

Reviewing answers

Even though the models were asked to answer with the predicted class only, often they replied with a long answer justifying their decision. For example, Llama-3.2 answered:

Considerations:
*1. **Nucleus Appearance**: The image shows cells with dark-stained nuclei. The shape and segmentation of nuclei are important for identifying white blood cell types.*
*2. **Cytoplasm and Granules**: Observe the characteristics of the cytoplasm and whether it contains granules or not. Basophils and eosinophils typically have granules, but their color and dispersion differ.*
*3. **Cell Size and Shape**: Consider the overall size and shape of the cells. Monocytes are generally larger with indented nuclei, while lymphocytes are smaller with a round nucleus.*

*4. **Granule Color**: The color and size of granules can help distinguish between eosinophils, which have large red-orange granules, and basophils, which have bluish-purple granules.*
*5. **Nuclear Segmentation**: Neutrophils have segmented nuclei, while blasts have larger, less segmented nuclei.*
Based on these considerations, the cells in the image display characteristics consistent with basophils, such as a lobed nucleus obscured by dark granules.

To extract the model's predicted class, we used GPT-4o with the following prompt:

```
"Chatbot answered: {chatbot's answer}
Which of the classes listed below does the chatbot's answer regarding
the cell type belong to? Write just the label (exactly as written below)
and nothing else:
      NA (Chatbot is unsure/ambiguous/doesn't know/no answer
provided/class cannot be determined)
      Band Neutrophil
      Basophil
      Eosinophil
      Erythroblast
      Lymphocyte
      Metamyelocyte
      Monocyte
      Myelocyte
      Platelet
      Promyelocyte
      Segmented Neutrophil"
```

Quantitative feature importance

To quantify the importance of morphological features for the model's decision-making, we used the following prompt:
"Consider the input image. Take a moment to think. Consider what features do the cells in the image have. Which of the white blood cell types listed below is shown? Write just the cell type and nothing else. Choose one of the possible labels provided below (exactly as written here): Band Neutrophil, Basophil, Eosinophil, Erythroblast, Lymphocyte, Metamyelocyte, Monocyte, Myelocyte, Platelet, Promyelocyte, Segmented Neutrophil.
Now consider the cell features listed below. Think how much each of them contributed to your cell classification decision that you made above. Next to each feature, write an importance score, how much the feature was important for your classification decision. The scores should be float numbers. All the scores together should sum to 100.
Cell Shape, Cell Size, Nuclear Shape, Nuclear Segmentation, Nuclear-to-Cytoplasmic Ratio, Nuclear Membrane Appearance, Nucleoli, Chromatin Pattern, Cytoplasmic Volume, Cytoplasmic Color, Cytoplasmic Border, Granule Presence, Granule Type, Inclusions (Presence of Auer rods, Döhle bodies, or other cytoplasmic inclusions), Cytoplasmic Basophilia, Erythrocytes, Platelets, Thrombocytes, Surrounding of the cell, Technical properties of the image (resolution, light, noise, etc.).

Are there any other features that you consider important for the classification decision? If yes, write them below."

Reasoning

We assessed the model's reasoning ability using the following prompt:

```
"Consider the input image. Take a moment to think. Consider what
features do the cells in the image have. Which of the white blood cell
types listed below is shown? Choose one of the possible labels provided
below (exactly as written here): Band Neutrophil, Basophil, Eosinophil,
Erythroblast, Lymphocyte, Metamyelocyte, Monocyte, Myelocyte, Platelet,
Promyelocyte, Segmented Neutrophil.
Explain in detail your decision and the reasoning that lead you to the
decision. Which parts of the cells and features did you consider? How
certain are you about your classification? Which other labels could be
correct? Why did you choose this label in the end?"
```

Computer vision

To test the model's segmentation abilities, we used the following prompts in sequence:

```
1. If present, highlight nucleus by painting in blue
2. Now, if present, highlight granules by painting in green
3. Now, highlight whole white blood cell in pink and all red blood
   cells in purple
```

Fine-tuning

OpenAI allows the user to choose the following hyperparameters when fine-tuning GPT-4o: batch size (BS), learning rate multiplier (LRM), number of epochs (NE). OpenAI does not let the user tune the learning rate directly, nor does it report what the actual value used was. The user can also choose that the model selects the hyperparameters automatically. Experimenting with the settings, we found that the automatic option usually gives the best result. The hyperparameter values used for the results presented in Fig. 3 in the main text were: at 1 image per class: NE=9, BS=1, LRM=2; at 5, 10, 25 and 50 images per class: NE=3, BS=1, LRM=2; at 100 images per class: NE=9, BS=6, LRM=2, at 200 images per class: NE=3, BS=4, LRM=2. The entries in the .jsonl files that we used for fine-tuning had the following form:

```
{"messages": [{"role": "system", "content": "You are an assistant that
identifies cell types."}, {"role": "user", "content": "Consider the
input image. Take a moment to think. Consider what features do the cells
in the image have. Which of the white blood cell types listed below is
shown? \n    Write just the cell type and nothing else. Choose one of
the possible labels provided below (exactly as written here):\n    Band
Neutrophil\n    Basophil\n    Eosinophil\n    Erythroblast\n
Lymphocyte\n    Metamyelocyte\n    Monocyte\n    Myelocyte\n
Platelet\n    Promyelocyte\n    Segmented Neutrophil"}, {"role": "user",
```

```
"content": [{"type": "image_url", "image_url": {"url":
"https://[...]/image_43.jpg"}}]}, {"role": "assistant", "content": "Band
Neutrophil"}]}
```

For fine-tuning DinoBloom-S and DINOv2, we appended a two-layer multilayer perceptron (MLP) with a ReLU activation between layers and a 128-unit hidden layer. During fine-tuning, we trained only the MLP while keeping DinoBloom-S or DINOv2 frozen. We used the Adam optimizer with a learning rate of 1e-3 and trained for 100 epochs, selecting the checkpoint with the lowest validation loss. Training and evaluation were conducted on an NVIDIA Tesla V100 (32 GB) GPU.

Paired bootstrap test

To statistically compare the performance of finetuned models, we performed a paired bootstrap analysis. We drew 20,000 different random re-samples of the test set. For each sample, we computed the differences in weighted F1 scores between two models:

$$\Delta_{wF1}(sample) = wF1_{model\ 1}(sample) - wF1_{model\ 2}(sample).$$

We then average Δ_{wF1} across all the samples to obtain $\bar{\Delta}_{wF1}$. We also compute 95 percentile confidence intervals of Δ_{wF1}. They represent the range of values that differences of weighted F1 scores could take in the worst case scenario if the test set was dramatically different. We also compute the bootstrap two-sided p-value:

$$p_{left} = mean(\{\Delta_{wF1}; \Delta_{wF1} \leq 0\})$$
$$p_{right} = mean(\{\Delta_{wF1}; \Delta_{wF1} \geq 0\})$$
$$p = 2 \cdot min(p_{left}, p_{right})$$

The bootstrap p-value measures the probability that the model with the lower mean F1 score performs better on some sample.

Here we show the statistical values for the comparison between the fine-tuned DinoBloom (model 1) and fine tuned GPT-4o (model 2) on the two data sets:

Supplementary Table 1. Paired bootstrap analysis between DinoBloom and GPT-4o models on Acevedo classification task

n	1	5	10	25	50	100	200
$\bar{\Delta}_{wF1}$	0.14	0.32	0.36	0.25	0.25	0.13	0.13
95-perc	[0.08,0.20]	[0.27,0.38]	[0.31,0.41]	[0.20,0.29]	[0.21,0.30]	[0.10,0.17]	[0.09,0.17]
p	$<10^{-4}$	$<10^{-4}$	$<10^{-4}$	$<10^{-4}$	$<10^{-4}$	$<10^{-4}$	$<10^{-4}$

Supplementary Table 2. Paired bootstrap analysis between DinoBloom and GPT-4o models on MLL23 classification task.

n	1	5	10	25	50	100	200

$\bar{\Delta}_{wF1}$	-0.04	0.02	0.06	0.02	0.17	0.14	0.09
95-perc	[-0.07,-0.01]	[-0.01,0.05]	[0.04,0.09]	[-0.01,0.05]	[0.14,0.20]	[0.10,0.16]	[0.06,0.12]
p	$2.6 \cdot 10^{-3}$	0.18	$<10^{-4}$	0.09	$<10^{-4}$	$<10^{-4}$	$<10^{-4}$

Note: in a 20000 sample bootstrap analysis, 10^{-4} is the smallest p-value interval that can be discriminated.

Random model

To establish a chance-level reference, we report the expected weighted F1 score of a stratified random classifier, which predicts each class independently with probability equal to its empirical frequency p_c in the test set. Under this model, predictions are independent of the ground truth, so the per-class recall reduces to p_c and the per-class precision reduces to p_c as well; substituting into the weighted F1 definition yields the closed form $wF1_{random} = \sum_c p_c^2$. This expression is computed directly from each dataset's test-set class frequencies. The same procedure is applied per attribute for the multi-attribute WBCAtt benchmark, with the final score averaged across attributes.

User experience

The first important information that we got when running the models was the user experience.

The best of all was GPT-4o. The website interface is transparent and easy to use. After obtaining an API key and topping up the account, the model ran smoothly and fast. A downside of GPT-4o is that the online instructions are scarce and chatGPT has not been trained on the most recent information on GPT-4o API calling, so it cannot provide instructions.

Second best model in terms of user experience was Llama-3.2. Once it was set up, it ran smoothly. The downside is that it did not run on GPUs with less than 80GB of memory, in our case we needed an A100 GPU. It was still the slowest model of all of them. Few-shot evaluation was particularly slow, and it took around 12h for a dataset to complete. Another flaw that we noticed with Llama-3.2 was that it always gave a very long answer with all the reasoning explained, even if it was asked to provide a short answer with the cell class only.

The third in user experience was Gemini-2.0. The web interface is in our opinion confusing and hard to navigate. The model would sometimes interrupt the runs with “resources exhausted” message. This is because Gemini-2.0 limits the number of API calls per minute, with no clear information available about what the limits are. Increasing the sleep time between consecutive API requests to 5 s helped, but the runs would sometimes still get interrupted. So we ended up wasting a lot of time to rerun the Gemini-2.0 jobs.

LLaVA-Med and DeepSeek-VL2 required running on older versions of Cuda and PyTorch

and older versions of Conda environments that are not easy to reconstruct. This took a significant amount of time to set up. DeepSeek-VL2 and LLaVA-Med failed to produce sensible outputs in the few-shot setting—DeepSeek-VL2 generated erroneous text composed of random numbers and letters, while LLaVA-Med either gave no response or simply noted the presence of red blood cells and described their function. CONCH worked smoothly but produced underwhelming results. DinoBloom worked flawlessly, but we used it only for the fine-tuning benchmark.

Fine-tuning with GPT-4o requires preparing the data set in a form of a .jsonl file first and uploading the images to some web location. We found the instructions how to prepare the .jsonl files on the OpenAI website[43] too scarce, and it took a lot of trial and error to figure out how to prepare them correctly. Fine-tuning works in two steps. The user first uploads the data sets and after sets up the training job. The .jsonl files do get checked during the uploading but even if they get approved, there is often an error with the .jsonl file reported later while running the training. Some of our single cell images were rejected with an explanation that they “contain faces” or “contain captchas” which “violates Open AI” policies. The model creates and stores a few checkpoints during the training. It was slightly surprising that the checkpoints were not created in the steps when the validation loss was the lowest, but they seemed to be created a bit randomly. It was also not clear whether the final result model was taken to be the one with the lowest loss. Slightly surprising for a rather expensive service - fine-tuning on a dataset consisting of 1100 images for 10 epochs costs around 100€.

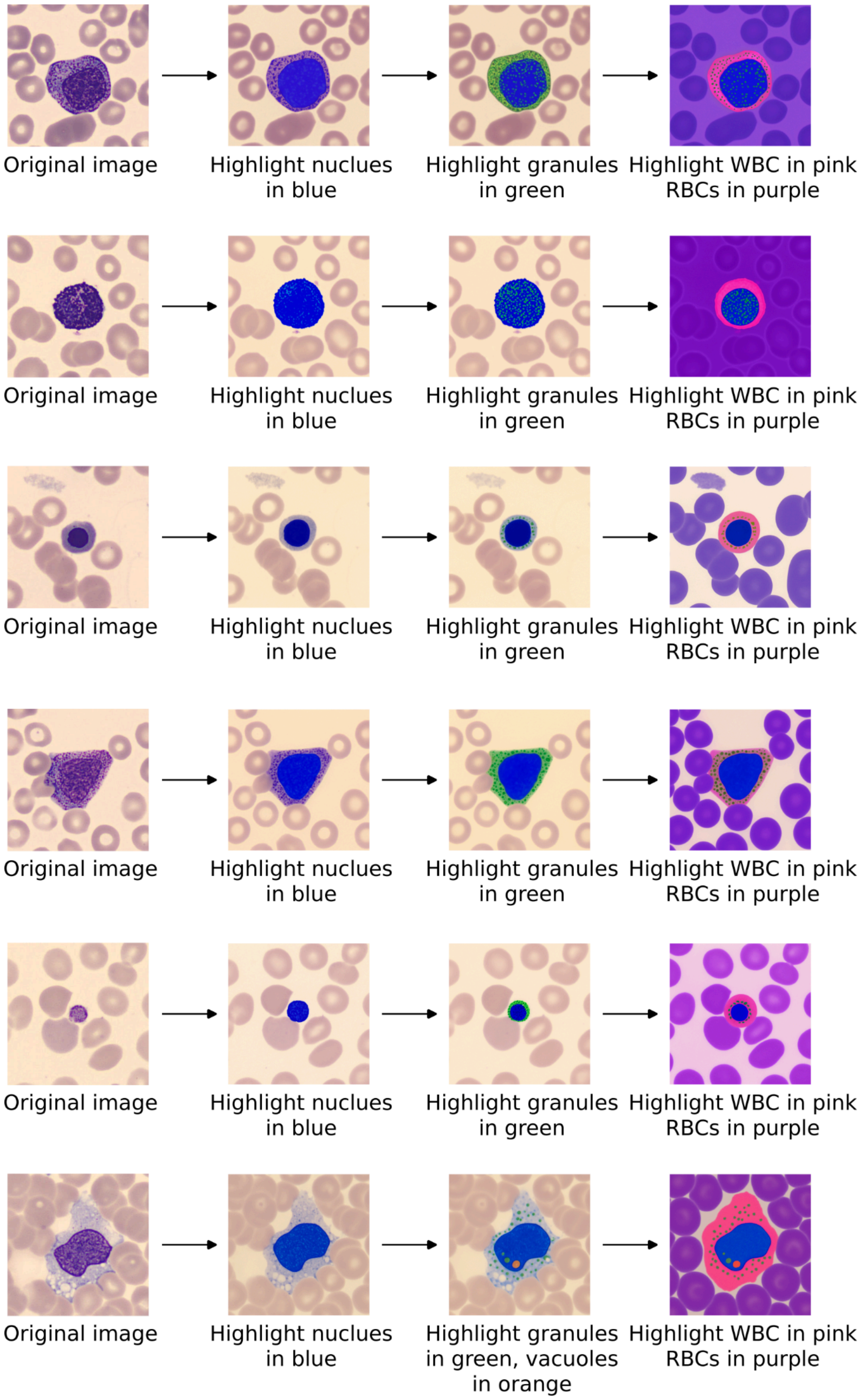

Supplementary Figure 1. Step-wise cell segmentation using GPT-4o. Although the model was prompted to segment nucleus and granules only if present, it consistently segmented certain components regardless of their presence, e.g. segmenting granules in monocytes, where they are typically absent. The nucleus was mostly segmented correctly, except in cases where it is not actually present, such as in platelets (third row). As expected, the model fails to segment the nucleus in basophils, where it is usually obscured by coarse granules. Otherwise, segmentations appear accurate when clear nuclei, granules, and red blood cell structures are present.

Supplementary Table 3. Blood cell type counts in peripheral blood smears as predicted by LVLMs. Responses are presented in their raw output format, including the units provided by the models.

**Cell Type**	**Reference** [44] [cells/mm^3]	**GPT-4o** [cells/mm^3]	**Gemini-2.0** [cells/mm^3]	**Llama-3.2** [cells/mm^3]	**DeepSeek-VL2** [mm^3]	**LLaVA-Med** [mm^3]
Promyelocyte	0	0	0	<1	0.0.00001 - 0.00005	2,345
Myelocyte	0	0	0	<1	0.0.01 - 0.0.5	2,859
Metamyelocyte	0	0	0	<1	0.0001 - 0.0005	2,071
Band neutrophil	0 - 700	0-600	0 - 700	100-700	0.01 - 0.05	10,765
Segmented neutrophil	1600-8000	1,500-8,000	1800 - 7800	1,500-8,000	0.0001 - 0.0005	37,910
Monocyte	100-700	100-800	200 -1000	200-1,000	0.0001 - 0.0005	3,129
Basophil	25-100	0-100	0 - 200	0-2	0.001 - 0.005	673
Eosinophil	50-500	0-500	0 - 500	0-600	0.005 - 0.05	2,957
Erythroblast	0	0	0	<1	0.0001 - 0.0005	3,268
Platelet	100,000-450,000	150,000-450,000	150,000 - 450,000	150,000-450,000	150.0 - 30.0	785,000
Lymphocyte	1000-4000	1,000-4,800	1,000 - 4,800	1,500-4,000	0.0.02 - 0.4	10,625
